# Interpersonal autonomy support style and its consequences in physical education classes

**DOI:** 10.1371/journal.pone.0216609

**Published:** 2019-05-20

**Authors:** Gracielle Fin, Juan Antonio Moreno-Murcia, Jaime León, Elisabeth Baretta, Rudy José Nodari Júnior

**Affiliations:** 1 Sports Research Center (CID), University Miguel Hernández, Elche, Alicante, Spain; 2 Department of Physical Education, University of West of Santa Catarina, Joaçaba, Santa Catarina, Brazil; 3 Department of Education, University of Las Palmas de Gran Canaria, Las Palmas de Gran Canaria, Las Palmas, Spain; University of Kentucky, UNITED STATES

## Abstract

This intervention study investigates the effects of teacher autonomy support on basic psychological needs, self-determined motivation for giving physical education classes and satisfaction from engaging in physical activity. The sample consisted of 61 students (32 in the experimental group and 29 in the control group), aged 12 to 14 years. Two physical education teachers were part of the group, one who was trained to give autonomy-support classes and the other used the usual class model. The experimental group teacher gave classes based on the autonomy support style, while his control group counterpart did not follow any model. The students, assessed before and after the 8-month intervention, were measured for perception of interpersonal teaching style, basic psychological needs, self-determined motivation and satisfaction from engaging in physical activity. The results showed that the experimental group exhibited higher indices for autonomy, competence and relatedness, self-determined motivation and satisfaction from engaging in physical activity, when compared to the control group. The study provides evidence of the effectiveness of programs that support autonomy in physical education classes, emphasizing the importance of pedagogical strategies and educational programs that promote the development of basic psychological needs, self-determined motivation and its positive consequences in relation to physical education classes.

## Introduction

The practice of exercises and physical activity and its benefits are clear; furthermore, people know that, generally, it is very important to have an exercise routine in order to maintain the physical abilities and health, once researchers substantiate the relation between the standard practice of guided physical education and health from childhood to adulthood [1].

During childhood and adolescence, the physical education classes offered in schools may be the ideal environment to provide the incentive and stimulus to the practice of physical and sportive activities. Researchers interested in optimizing the youth motivation in the context of School Physical Education have focused their interests on understanding the several motivational processes that determine the involvement levels in Physical Education or in any other sports context [2].

Despite the consensus among experts that including physical education as a mandatory component of school curricula is vital to the process of encouraging children and adolescents to engage in physical activity, adherence and promotion of the health-related benefits has been unsatisfactory [3]. Interventions to awaken the interest and participation of elementary school students in physical education classes are importants in order to disseminate scientific knowledge to teachers and facilitate their practice [4].

Given that physical education classes should include interventions that encourage physical activity from childhood to adolescence, researchers interested in optimizing young people’s motivation in the context of physical education in schools have concentrated their efforts on understanding the different motivational processes that determine the levels of involvement in these activities [2]. A number of studies have highlighted the need for intervention programs involving teaching techniques and student engagement as an effective strategy to increase the latter’s motivation to engage in physical education [5–8]. To that end, a theoretical framework, such as the self-determination theory (SDT), is needed to explain student behavior [9].

### Motivation and interpersonal style of physical education teachers

The SDT, proposed by Deci and Ryan [9–10], aims to explain human behavior, based on different motivational styles, influences of the context, and interpersonal perceptions. Three basic psychological needs are related to motivation: autonomy, which is related to the level of independence and control of the choices made by an individual; competence, which refers to a person’s ability to perform a task; and relatedness, which is linked to the perception of a sense of connection with other people [11].

Motivation is understood as a continuum of three types of motivation, varying from the most self-determined form (intrinsic motivation) to the lowest self-determination levels (extrinsic motivation and amotivation) [9–10]. The intrinsic motivation is the highest level of self-determination, in this case the choice is personal, characterizing total autonomy in terms of the activity, which generates interest, pleasure and satisfaction inherent to the activity. An intrinsically motivated person exhibits feelings of competence and self-accomplishment, sustaining interest for the activity even after the goal has been achieved [9–10]. More self-determined styles would be associated with pleasure, the effort to perform the activities and the perception of a context favorable to autonomy, while less self-determined styles would be associated with anxiety and discomfort in carrying out these activities.

Extrinsic motivation is determined by four types of regulation and their regulatory processes: external regulation is characterized by the behavior to attain a desired consequence such as tangible rewards or to avoid a threatened punishment; in introjected regulation the rewards involved in the regulatory process are internal, individuals feel that they “need” or “must” perform a certain activity, but there is no feeling or “wanting” to do it; the regulation identified is more internally regulated behavior, in this case individuals consider their participation in the activity important; integrated regulation is considered the most self-determined of the extrinsic motivations, for it not only involves identifying with the importance of behaviors but also integrating those identifications with other aspects of the self [10].

Amotivation represents the lack of both types of motivation and thus a complete lack of self-determination with respect to the target behavior. The need to perform an activity will not be valued, and will be accompanied by feelings of frustration, incompetence and fear [10].

SDT [10,11] has been used as the conceptual framework in numerous studies related to physical activity and physical education classes, given that teacher interpersonal style may influence its effects student motivation, commitment and learning [12–14].

One of the principles of SDT is that teacher interpersonal style influences the motivation of students during physical education classes, and may characterize extreme control or significant autonomy support [15]. A teacher style aimed at autonomy will meet the basic psychological needs of competence, autonomy and relatedness, thereby reaching the inner motivational resources of the students, providing explanations and allowing them to learn at their own pace without using controlling language [16]. On the other hand, a controlling style is characterized by controlled and hostile behavior, resulting in classes that mirror the teacher’s way of thinking, feeling and behaving, leading to external motivational resources, given that students receive encouragement contingent with results, threats or punishment. The students disregard their own inner motivational resources, concerning themselves with meeting the needs of the teacher when engaging in any activity [17]. The neutral style may be determined when the professors put themselves in a position of lack of interest, in other words, there is neither relation of support to the autonomy nor relation of control during the classes.

An autonomy-based teaching style has been related to positive results, since it improves student performance, makes them feel more competent to execute activities and more persistent in achieving good outcomes. This causes a positive change in behavior in the face of a proposed objective, either during class goals [18–19] or in terms of health and well-being-related [20].

Despite the large number of studies indicating that teaching strategies aimed at autonomy support improve the quality of interactions with students, favoring and enhancing positive experiences during class [21], teachers tend to use a controlling style [22]. Thus, it is important to conduct studies that analyze teacher behavior, examining whether changes are needed and if these changes influence student behavior in physical education classes.

### Intervention studies on the interpersonal style of teachers and motivation

The fundamentals of SDT have been applied in intervention studies aimed at demonstrating that changes in teacher behavior influence the motivational profile of students. When teachers adopt an autonomy support style, it has resulted in benefits in terms of the physical activity of their students [8]. However, intervention programs have shown to be more efficient when teachers are trained on the topic, emphasizing not only theoretical content, but also the application of SDT practices, in addition to autonomy-based classes [23,24]. As such, there is a need for studies that promote teacher training for this type of class.

Intervention studies have been conducted in physical education classes to examine the effect of autonomy support on self-determined motivation and to meet the basic psychological needs of the students, resulting in positive behaviors [8,12,25,26].

The studies show the effect of the intervention on student involvement in class, observing their emotional and cognitive behavior, but only one [8] observed a relation with physical activity level. These studies also used a restricted approach regarding the effects of the intervention, given that either self-determined motivation or basic psychological needs were observed. Complementary dimensions can influence motivation and it is important to consider teacher interpersonal style aimed at autonomy or control of the three basic psychological needs (autonomy, competence and relatedness), and the feeling of satisfaction with physical education classes.

### The present study

The aim of this intervention was to assess whether an 8-month autonomy support program had an effect on the basic psychological needs (autonomy, competence, and relatedness) and self-determined motivation of students to attend physical education and their satisfaction from engaging in physical activity.

The methodology of the current study is similar to that of previous studies that have been performed on this topic [12, 17, 21, 24, 27–30]. It was conducted with elementary school students, divided into an experimental group that followed the autonomy support teaching model and a control group that did not follow any established model. Each group had a different teacher; the experimental group’s teacher used an autonomy-based style, while the control group received no intervention.

The hypothesis was that the autonomy support group would benefit from the intervention and exhibit better autonomy, competence and relationship levels, greater self-determined motivation for physical education classes and more satisfaction from engaging in physical activity, compared to their control group counterparts.

## Methods

### Participants

The sample consisted of sixty-one 7^th^ grade students enrolled in a Brazilian public and urban school, aged between 12 and 14 years (*M =* 12.9, *TD* = .69). Participants were divided into an experimental group (*n* = 32), consisting of 19 girls and 13 boys, and a control group (*n* = 29), with 14 girls and 15 boys. The percentage distribution by sex was 54% for the girls (*n* = 33) and 46% for the boys (*n* = 28). Two physical education teachers were part of the group, one who was trained to give autonomy-support classes and the other used the controlling style model.

### Measures

#### Autonomy support

The Autonomy Support Scale (ASS), created by Moreno-Murcia, Huéscar, Fabra and Sánchez-Latorre [31], validated for Brazil by Fin, Moreno-Murcia, Baretta and Nodari Júnior [32], is composed of 11 items, which, using a single factor, measure student perception of autonomy support offered by teachers in physical education classes. The items (e.g. “His explanations help us understand the purpose of the activities we engage in”) are preceded by the statement “In my physical education classes, my teacher …”. All the answers correspond to a Likert-type scale that varies from 1 (*Strongly disagree*) to 5 (*Strongly agree*). A Cronbach’s alpha for pre- and post-test collection of .91 and .94, respectively was obtained.

#### Controlling style

The Controlling Style Scale (CSS), created by Moreno-Murcia et al. [31], was validated for Brazil by Fin et al. [32]. The Brazilian version contains 7 items that measure student perception of the controlling style of physical education teachers. The items (e.g. “Speaks continuously and does not allow us to contribute to the class”) are preceded by the statement “In my physical education classes, my teacher …”. All the answers correspond to a Likert-type scale that varies from 1 (*Strongly disagree*) to 5 (*Strongly agree*). A Cronbach’s alpha for pre- and post-test collection of .76 and .89, respectively was obtained.

#### Basic psychological needs

A questionnaire was applied to assess basic psychological needs in physical education (NPBEF), adapted for Portuguese by Pires, Luís, Borrego, Alves, and Silva [33] from the Basic Psychological Needs in Exercise Scale (BPNESp) [34]. The questionnaire consists of 12 items encompassing three dimensions: autonomy (e.g. “I feel I do activities the way I want to”), competence (e.g. “I feel I complete class activities successfully”), and relatedness (e.g. “I feel good with my classmates”). Items are preceded by the stem “Generally, in physical education …” and are scored on a 5-point Likert scale from 1 (Completely disagree) to 5 (Completely agree). Cronbach’s alpha was .87, .62, .73, respectively for pre-test and .95, .82, .67 for post-test.

#### Motivation

The Perceived Locus of Causality Questionnaire (PLOCQ) [35] was used, translated into Portuguese and validated for the Brazilian population [36]. The questionnaire contains twenty items and is subdivided into five dimensions: intrinsic motivation (e.g. “Because physical education is fun”); identified regulation (e.g. “Because I want to learn sports skills”); introjected regulation (e.g. “Because I want the teacher to think I am a good student”); external regulation (e.g. “Because I am supposed to do it”); amotivation (e.g. “But I really feel I am wasting my time”). Items are preceded by the stem “I do physical education …” and are scored on a 7-point Likert scale ranging from 1 (Completely disagree) to 5 (Completely agree). Internal consistency was .79, .70 .62, .66 e .61, respectively for pre-test and .67, .76 .75, .74 e .60, for post-test. As scores obtained in each of the dimensions of the PLOC to know the index of self-determination (IAD): (2 x intrinsic motivation + identified regulation)–[(introjected regulation + external regulation) / 2 + 2 x amotivation]) [37]. In this study the index was –5.50 and 15.40 (*M* = 7.26, *DP* = 4.16) for pre-test and -5.30 and 15.40 (*M* = 8.01, *DP* = 3.88) for post-test.

#### Physical activity enjoyment scale

We applied the Physical Activity Enjoyment Scale (PACES) [38], translated by Montanha [39], to measure enjoyment of physical activity. The scale consists of 16 statements preceded by the stem “When I am physically active …,” which assess enjoyment directly (e.g. “I enjoy it,” “It’s very pleasant,” “It gives me energy”) and inversely (e.g. “It makes me sad,” “I dislike it,” “It’s no fun at all”). Answers were scored on a Likert-type scale, rated from 1 (Completely disagree) to 5 (Completely agree). Cronbach’s alpha was .91 for pre-test and .89 for post-test.

#### Teacher interpersonal style in physical education

The measurement scale for teacher interpersonal style in physical education (MEIDEF) [40] was used to assess teacher interpersonal style during intervention classes. This behavior observation scale of teacher interpersonal style in autonomy support (AS), controlling style (CS) and/or neutral style (NS) consists of 60 items preceded by the statement “when the teacher assigns a task”, grouped into one construct that considers four dimensions: 1) autonomy, with five items for AS (e.g., “Asks the students about their preference in relation to the task”) and five for CS (e.g., “Does not ask or consider student preferences”); 2) pre-task structure, with five items for AS (e.g., “Explains and presents the objectives at the start of the class”) and five for CS (e.g., “Does not explain the objectives and stays focused on the content”); 3) structure during the task, with eight items for AS (e.g. “Adapts instructions according to the students’ progress”), eight for CS (e.g. “Provides constant information, regardless of progress (using controlling language”) and four for NS (e.g., “Provides information without encouraging progress or using controlling language”); and 4) relatedness, with seven items for AS (e.g., “Uses empathetic language”), seven for CS (e.g., “Does not use empathetic language and does not adapt to the students”) and five for NS (e.g., “Uses apathetic language when addressing the students, with no emotion or comments, positive or negative)”. The observations are divided into tasks, whose number varies depending on the type of class proposed by the teacher.

### Study procedure and design

The investigation was approved by the Human Ethics Committee of the University of Western Santa Catarina (Unoesc)–Brazil, under protocol number 1.977.830, of March 22, 2017. The chief investigator contacted the school principal to present the general objectives and procedures of the study. Next, parents or caregivers gave their informed written consent for the students to answer the questionnaires and be filmed for subsequent analysis. Consent was obtained from all those responsible and all students were able to participate in the study. All the participants were treated in accordance with the institutional ethical guidelines, respecting the consent, confidentiality and anonymity of the responses.

Sample selection resulted in a quasi-experimental design [41], given that participants could not be randomly selected, since the students were allocated to pre-determined groups.

Two of the four 7^th^ grade classes were randomly selected. The experimental group had a teacher followed an autonomy support teaching model, while the control group had a teacher followed a controlling style model.

To reduce possible risks, neither group was told the study objectives. Students were told that they would participate in a study that required anonymously filling out questionnaires, in order to assess the school’s physical education classes. They were also informed that the physical education classes would be filmed to observe teacher behavior.

**Fig 1:**
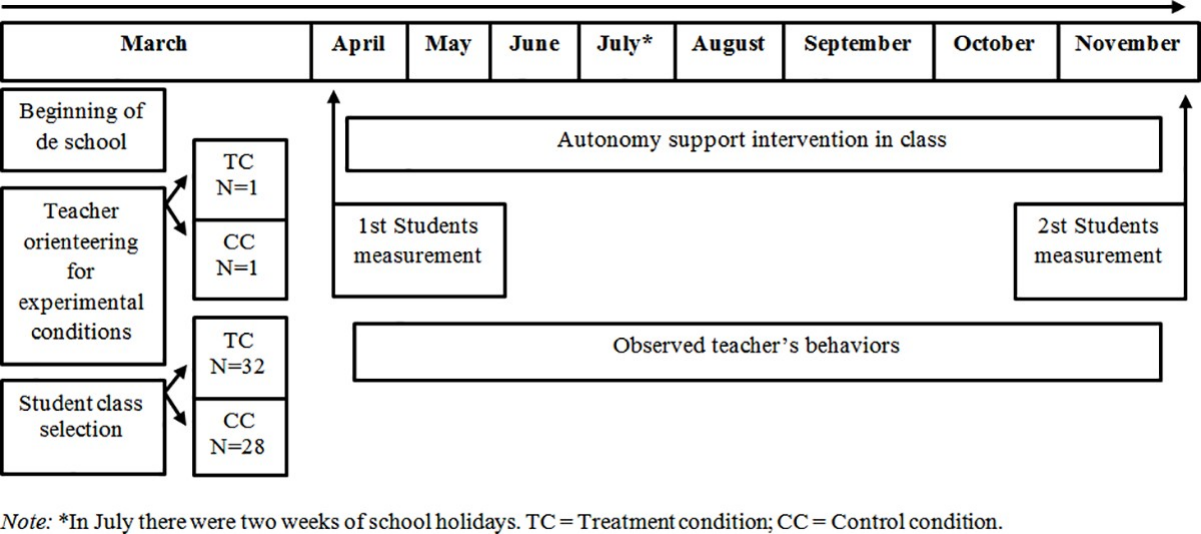
Procedural timeline for the autonomy support intervention and data collection. *Note*: *In July there were two weeks of school holidays. TC = Treatment condition; CC = Control condition.

The questionnaires were completed in approximately 35 minutes by both groups during class time, under supervision of the researcher. Data collection took place at the start of the school year, before the intervention and nine months later, at the end of the school year (Fig 1).

Before the intervention, the experimental group teacher was trained in the interpersonal autonomy support style. To that end, forty hours training seminars were held to explain and discuss how to organize and give classes using an autonomy support style, based on existing models [17,21,24,27–30]. Seminars on SDT [42], and the Hierarchical Model of Intrinsic and Extrinsic Motivation (HMIEM) [43,44] were also held. Strategies proposed in the literature were also analyzed to implement the autonomy, controlling and neutral styles. The interventions of the professor who followed a model based on autonomy involve allowing the student make bigger decisions during the task, with bigger resolution of problems. In order to increase the support to autonomy in physical education classes, the professor needs to present a series of characterstics, such as: demonstrate interest in teaching and in the students’ learning; be positive; be patient and listen to the students; give more importance to the class process than to the final product; respect the differences among the students, their learning paces, behaviors and interest; demonstrante empathy and manage the emotions well during conflicts [16,17,22,24,29,30,45,46].

**Fig 2:**
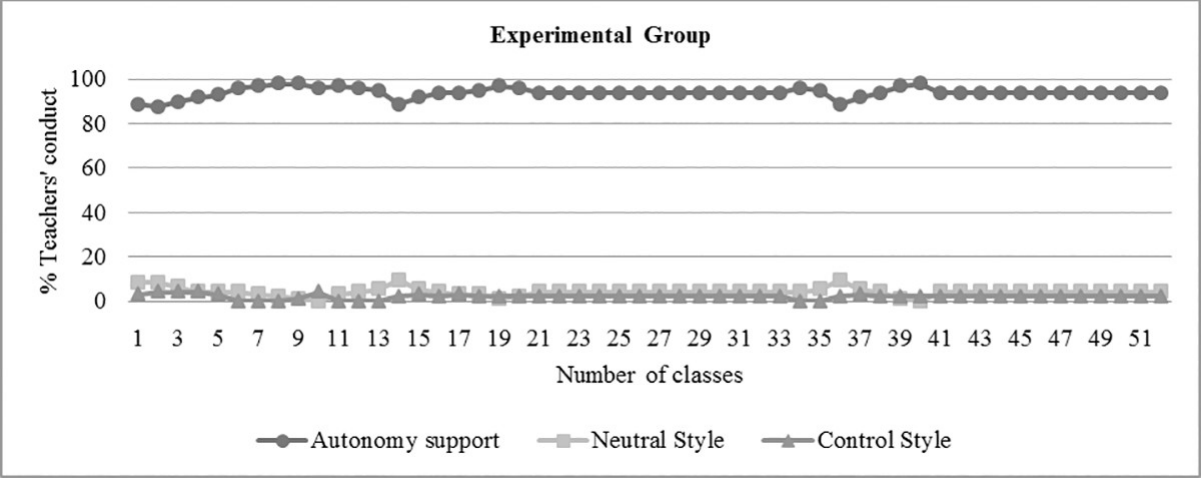
Evolution of teacher interpersonal style percentages (Autonomy Support, Control Style, Neutral Style) in the experimental group.

**Fig 3:**
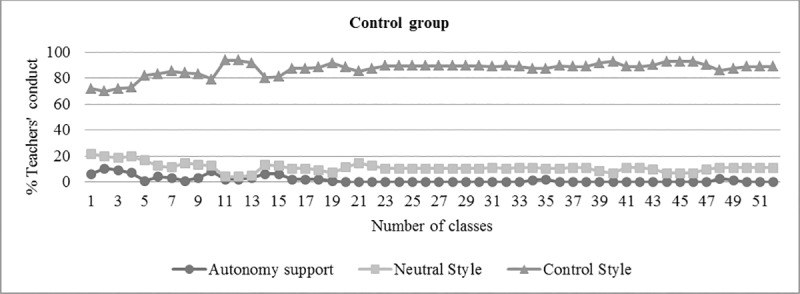
Evolution of teacher interpersonal style percentages (Autonomy Support, Control Style, Neutral Style) in the control group.

After teaching training, two classes were filmed in order to gather evidences of the teacher interpersonal style. One of the tasks was a separate analysis by both the chief investigator and the experimental group teacher. The teacher was able to demonstrate his competency and understanding of these models, as demonstrated in the data analysis (Table 1, Fig 2 and Fig 3).

**Table 1:** 

	Treatment condition		Control condition	
	Frequency	%	Frequency	%
Autonomy Support	4683	94%	84	2%
Control Style	100	2%	3940	87%
Neutral Style	207	4%	512	11%
Total	4990	100%	4536	100%

The intervention was conducted between April and November 2017 (except during the July school holidays), with a total of fifty-two 55-minute classes, on a twice weekly basis. It is important to underscore that the students had to walk from the classroom to the gym, which usually took 15 minutes (back and forth), thereby reducing class time to about 40 minutes. To assess and control the intervention, one physical education class per week of each group was filmed between April and November 2017.

### Data analyses

Descriptive analyses were carried out to evaluate teacher interactions during the classes, using the measurement scale for teacher interpersonal style in physical education. According to some studies [12,21,22,45], that took similar measurements, 80% or more of the interactions recorded using the teacher interpersonal style should be aimed at autonomy of the experimental group. On the other hand, in the control group, 80% of the interactions should be characterized by the controlling style. In the present study, both groups obtained indices within those reported in the literature, as shown in Table 1, Fig 2 and Fig 3. Kappa’s coefficient of interrater agreement was .87 and .93.

**Fig 4:**
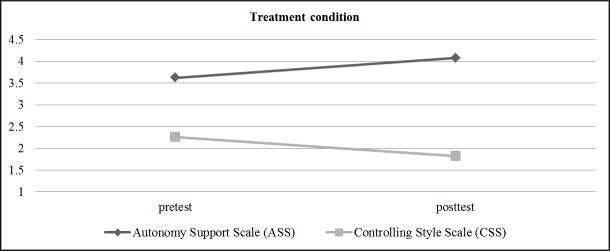
Effect of the evolution of teaching interpersonal style (Autonomy Support Scale, Controlling Style Scale) on the experimental group.

**Fig 5:**
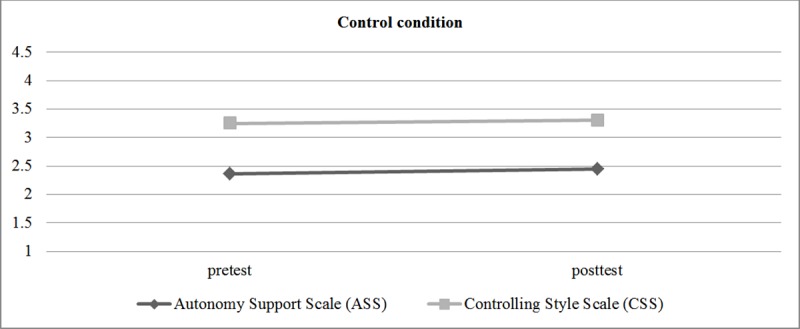
Effect of the evolution of teaching interpersonal style (Autonomy Support Scale, Controlling Style Scale) on the control group.

To obtain the perspective of students regarding the effects of the intervention, after covariance tests were performed, the intervention effect on perceived autonomy support was measured using the Autonomy Support Scale (ASS) and Controlling Style Scale (CSS) (Fig 4 and Fig 5). Differential analysis was conducted and the results show the intervention effect on autonomy support in the experimental group (*M*_Measure 1_ = 3.62 and *M*_Measure 2_ = 4.08, *p* < .001) as well as controlling style (*M*_Measure 1_ = 2.26 and *M*_Measure 2_ = 1.82, *p* < .001), obtaining an increase in perceived autonomy and a decline in perceived control. The control group showed no differences in perceived autonomy (*M*_Measure 1_ = 2.36 and *M*_Measure 2_ = 2.45) or controlling style (*M*_Measure 1_ = 3.25 and *M*_Measure 2_ = 3.31).

### Statistical analysis

Descriptive analyses were conducted to confirm the teacher interpersonal style and Kappa’s coefficient to establish agreement between the investigator and the professor evaluated. Descriptive analyses and multivariate (MANOVA) and univariate (ANOVA) analyses of variance were applied to determine pre- and post-test group scores. Next, descriptive and post-test analyses of covariance (MANCOVA and ANCOVA post-test) were conducted. Finally, descriptive and pre and post-test analyses of covariance (MANCOVA and ANCOVA pre-post-tests) were carried out. Internal consistency tests were also performed (Cronbach’s α: desirable > .70, satisfactory > .60-.70) [47] and the effect size calculated (Cohen's d: small < .50, moderate = .50-.79, large ≥ .80) [48] for each variable, in the pre-test, post-test and pre-post-test differences. Statistical analyses were conducted using the SPSS 20.0 program.

## Results

### Effects of autonomy-based intervention

Multivariate analysis of the pre-test data obtained for the groups was applied to assess the effects of teacher autonomy support during physical education classes on the variables self-determination, basic psychological needs and satisfaction from engaging in physical activity. The results of pre-test MANOVA (Table 2) (Wilk’s Lambda, Λ = 0.09, *F* (13, 47) = 34.09, *p* < .00) revealed a difference in autonomy and competence for the experimental group, and intrinsic motivation, identified regulation, introjected regulation and lack of motivation for the control group.

**Table 2:** 

	Pretest ANOVA			Posttest ANCOVA			Posttest-pretest ANCOVA		
	F	*p*	D	F	*P*	d	F	*P*	d
Autonomy	64.77	.00	.52	211.17	.00	.88	79.70	.00	.72
Competence	23.84	.00	.28	75.48	.00	.55	13.97	.00	.18
Relatedness	1.86	.18	.14	43.71	.00	.59	32.87	.00	.35
Intrinsic motivation	4.89	.03	.06	229.12	.00	.88	19.92	.00	.24
Identified regulation	7.62	.01	.10	676.90	.00	.96	1.26	.27	.00
Introjected regulation	4.77	.03	.06	159.83	.00	.84	12.65	.00	.16
External regulation	.89	.35	.00	73.37	.00	.71	29.99	.00	.33
Demotivation	4.80	.03	.06	158.76	.00	.84	1.53	.22	.01
SDI	.41	.53	.01	372.26	.00	.93	57.65	.00	.49
Enjoyment	2.56	.12	.03	7.54	.00	.18	13.42	.00	.17

Note: SDI = Self-determination index

The post-test MANCOVA test scores revealed intergroup differences (Wilk’s Lambda, Λ = 0.039, *F* (13, 47) = 89.81, *p* < .00), with the experimental group exhibiting higher autonomy, competence, relationship and self-determined indices and more satisfaction from attending physical education classes, in addition to lower identified, introjected and external motivation values, and lack of motivation (Table 3).

**Table 3:** 

	Pretest				Posttest				Posttest-pretest			
	Treatment condition		Control condition		Treatment condition		Control condition		Treatment condition		Control condition	
	*M*	*SD*	*M*	*SD*	*M*	*SD*	*M*	*SD*	*M*	*SD*	*M*	*SD*
Autonomy	3.23	.75	1.80	.62	4.29	.29	2.03	.57	1.07	.67	.23	.76
Competence	3.90	.54	3.14	.68	4.33	.36	3.11	.51	.43	.44	-.02	.50
Relatedness	3.84	.59	4.07	.72	4.37	.34	3.93	.73	.53	.53	-.13	.35
Intrinsic motivation	4.69	1.16	5.35	1.15	5.15	.93	5.36	1.14	.46	.50	.01	.23
Identified regulation	4.57	1.10	5.34	1.08	4.72	1.22	5.57	1.17	.15	.21	.23	.32
Introjected regulation	3.53	1.00	4.23	1.48	3.15	1.09	4.36	1.37	-.38	.74	.13	.24
External regulation	3.22	1.96	3.66	1.61	1.66	.48	4.07	1.58	-1.56	1.58	.41	1.18
Demotivation	1.81	.54	2.24	.95	1.92	.69	2.45	.87	.11	.34	.21	.31
SDI	6.94	4.41	7.62	3.92	8.77	3.80	7.18	3.86	1.83	1.34	-.44	.94
Enjoyment	3.73	.62	4.00	.73	4.23	.44	3.83	.63	.50	.66	-.18	.79

Note: SDI = Self-determination index

We analyzed pre-post-test changes in order to assess intervention effectiveness. The pre-post-test MANCOVA results showed differences in the variables as a whole (Wilk’s Lambda, Λ = 0.024, *F* (26, 34) = 53.31, *p* < .00). Comparison of the changes found in pre-post-test intergroup results demonstrated a decrease in introjected and external motivation scores in the experimental group, and an increase in autonomy, competence, relatedness, intrinsic motivation, self-determined index, and satisfaction from engaging in physical activity. The control group showed a decline in competence, relatedness, self-determined index and satisfaction from attending physical education classes, and an increase in the remaining items, but no difference in identified regulation and demotivation.

## Discussion

This study aimed at assessing the effects of teacher autonomy support on autonomy, competence, relatedness, self-determined motivation for attending physical education classes and satisfaction from engaging in physical activity of students.

### Teacher interpersonal style during physical education classes

As expected, the teacher of the experimental group increased or maintained the use of autonomy support during his classes, sustaining a higher level of involvement over time when compared to the control group teacher. The results corroborate earlier studies, given that the use of a teacher autonomy support style favors improvements in the motivational aspects of the students, but also reflects in the profile of the teachers themselves, causing the same structure of clear and consistent goals to decrease control, which favors better interactions and, in turn, enhanced feelings of satisfaction in the teachers [8].

The social context where activities are practiced has a significant impact that can favor or frustrate the basic psychological needs and a feeling of satisfaction from attending physical education classes [10]. When physical education classes are viewed as a specific social context [49] the extent to which teachers influence student motivation becomes clear [12]. As such, the more teachers master autonomy support and use these strategies during their classes, the greater the student commitment, the higher their levels of autonomy, competence, relatedness, self-determined motivation, satisfaction from engaging in physical activity and consequently, the more physically active they will be.

Educational autonomy support strategies that stimulate the development of self-determined motivation may lead students to become involved in regular physical activities, since they are less likely to discontinue them at the end of the school year. It is important to underscore the relation between feelings of fulfillment and pleasure during physical activity and student adherence [3,50].

### Effects of intervention on the students

The results obtained confirm that the intervention has a positive effect, since students who received greater autonomy support increased their scores in the following domains: basic psychological needs, intrinsic motivation, self-determined index and satisfaction from attending physical education classes. The control group scores declined with respect to competence, relatedness, self-determined index and satisfaction from engaging in physical activity. These results are consistent with other studies showing the effectiveness of teacher interventions that increase autonomy support during class [8,51].

The explanations for the results of this study may be related to the characteristics of the intervention conducted in the physical education classes, such as varying activities, transmitting a feeling of responsibility, allowing students to make decisions, recognizing effort and personal improvement, and emphasizing self-determined motivation. Autonomy support produced positive results for basic psychological needs, self-determined motivation and satisfaction from engaging in physical activity [13,50], since it was confirmed that students who received greater teacher autonomy support were also more likely to participate in proposed tasks, exhibit greater commitment to their activities and perceived competence, in addition to being more satisfied with their lives [52].

## Limitations and conclusions

This study showed that an intervention program aimed at autonomy can benefit students, but like other studies, it also has limitations. A method is needed to assess the amount of exercise performed or motor improvement in physical education class activities. The small sample may also be a limiting factor, since it highlights the difficulty in recruiting teachers to participate in intervention studies.

It is suggested that future studies control lesson plans and consider the curricular aspects of physical education, in order to determine the program content differences over the course of the intervention. Furthermore, to determine the permanence or stability produced by the intervention, future investigations should include a one-year post-intervention follow-up. There is also a need to systematize teacher strategies for physical education classes aimed at autonomy support.

In conclusion, the study provides evidence of the effectiveness of autonomy support programs in physical education classes. The results emphasize the importance of devising pedagogical strategies and educational programs that favor autonomy during adolescence in order to develop their basic psychological needs and self-determined motivation. The feeling of being satisfied with physical education classes results from more self-determined behavior and fulfilment of the basic psychological needs of competence, autonomy and relatedness, which are influenced by teacher interpersonal style.

## Supporting information

S1 File Database. (SAV)
